# Mindfulness-Based Cognitive Therapy–Game: An Ironic Way to Treat Internet Gaming Disorder

**DOI:** 10.2196/65786

**Published:** 2025-03-27

**Authors:** Jaehyun Kim, Hayoung Oh, Anderson Sungmin Yoon

**Affiliations:** 1 Department of Applied Artificial Intelligence College of Computing and Informatics Sungkyunkwan University Seoul Republic of Korea; 2 College of Computing and Informatics Sungkyunkwan University Seoul Republic of Korea; 3 Department of Social Welfare Sungkyunkwan University Seoul Republic of Korea

**Keywords:** cognitive behavior therapy, psychosocial intervention, video games, internet gaming disorder, internet addiction, mindfulness, mental health

## Abstract

Internet gaming disorder (IGD) affects 3% of the global population and poses an increasing risk due to advancements in technology. However, there is currently no definitive treatment for this condition. IGD is not a primary disorder but rather a result of “self-prescription” in response to emotional stressors. Unlike conventional mental health treatments that focus on the disorder itself, it is crucial to provide alternative activities that can alleviate negative emotions. This paper extends the concept of the self-medication hypothesis and integrates it with cognitive models of cognitive behavioral therapy and mindfulness-based cognitive therapy. In addition, it introduces the mindfulness-based cognitive therapy–game (MBCT-G), a program designed to explore alternative activities through gaming, focusing on the processes of response and reward, which are not typically emphasized in traditional treatments. This study serves as the theoretical foundation for the development of MBCT-G. MBCT-G aims to train individuals in positive coping strategies that alleviate psychological distress, offering a novel approach to treating self-prescription disorders such as IGD.

## Introduction

The prevalence of internet gaming disorder (IGD) is known to be around 2% to 3% [1]. This prevalence varies depending on the region and the population studied [2-4]. In 2013, the American Psychiatric Association introduced IGD as a new disorder in the *Diagnostic and Statistical Manual of Mental Disorders, Fifth Edition* (*DSM-5*). The World Health Organization (WHO) also recognized gaming disorder as a disease in the *International Statistical Classification of Diseases and Related Health Problems, 11th Edition* (*ICD-11*). However, although *DSM-5* provides standard definitions and diagnostic criteria, systematic treatment methods for gaming disorders have not been thoroughly reviewed, and clinical evidence remains insufficient [5,6]. Currently, cognitive behavioral therapy (CBT) is the most frequently used treatment method; however, it has not been proven superior to other treatments [7]. Patients with gaming disorder tend to prioritize gaming above all else, lose control over their gaming behavior, and exhibit an inability to voluntarily stop [8]. While these clinical features are similar to those of substance use disorder (SUD), the two conditions show significant differences from a therapeutic perspective.

Moreover, the term “internet gaming disorder” is often confused with other conditions. For instance, pathological internet use (PIU) refers to various online activities, such as gaming, social networking, shopping, watching videos, and reading newspapers that interfere with daily life. Some studies on IGD often include PIU, which is not entirely identical to IGD [9]. According to one study, less than half of the patients with IGD met the criteria for PIU, while only 6.67% of those who met the criteria for PIU were classified as having IGD [10].

IGD possesses characteristics that are difficult to explain within the existing psychiatric disorder framework. Traditional attempts to treat individuals addicted to internet games may be ineffective or, at times, may negatively impact the trust relationship with the patient. Therefore, this study proposes a hypothesis regarding the pathogenesis of IGD based on its characteristics. This hypothesis serves as a complementary system to the interacting cognitive subsystem (ICS) model, which has been theoretically used to explain CBT and mindfulness-based cognitive therapy (MBCT). In addition, the study discusses the necessity and potential development of mindfulness-based cognitive therapy–game (MBCT-G).

## Background

### Self-Medication Hypothesis and IGD

The self-medication hypothesis refers to the tendency of individuals to autonomously select substances or specific behaviors to alleviate psychological or emotional distress [11]. According to the self-medication hypothesis, patients experience temporary relief from psychological pain through substances like cocaine or opioids. The rewarding effect of such substances weakens patients’ self-control and increases their dependency on the selected substance. Patients who lose their self-control fail to consider the consequences of addiction, thereby heightening the likelihood of substance use and dependency [12].

In various studies on the causes and motivations behind IGD, the self-medication hypothesis has been discussed [13-17]. During pandemic situations like COVID-19, the prevalence of IGD increased [18,19], alongside a decline in self-control and a rise in depression and anxiety [20-22]. Resilience, defined as the ability to overcome stress, adversity, and psychological distress to return to a stable psychological state, tends to be lower in individuals with IGD, who also exhibit higher levels of depression [23,24]. Patients with posttraumatic stress disorder (PTSD), a representative anxiety disorder, are more likely to experience IGD, providing empirical support for the co-occurrence of these conditions [25,26]. While it remains unclear whether problematic gaming behavior is a cause or a consequence of weakened self-control, depression, and anxiety associated with COVID-19 and PTSD, the self-medication hypothesis is likely applicable to IGD, akin to its role in SUDs.

Research indicates that antidepressants like escitalopram and bupropion improve both IGD symptoms and depressive symptoms [27,28]. Such findings suggest that underlying causes may precede problematic gaming behavior. Previous studies have shown that bupropion is effective in managing attention and impulsivity, while escitalopram is effective in alleviating depressive symptoms. In particular, the findings from most studies suggesting that bupropion is more effective than escitalopram in improving symptoms of IGD [29,30] imply the necessity of managing the adverse effects of self-medication, such as reward effects of maladaptive behaviors and reduced self-control, regardless of the underlying causes driving problematic gaming behavior.

The similarities between IGD and SUD have been explored in various studies. Both conditions exhibit similar impulsivity traits [31], and neurological tests using functional magnetic resonance imaging have identified overlapping features [32]. However, individuals with SUD tend to have experienced more severe past adversities and exhibit more extreme problematic behaviors than those with IGD [33]. This distinction suggests that the two conditions may represent a continuum of self-medication choices, influenced by the severity of underlying causes. The high accessibility and immediate rewards of internet gaming may serve as a gateway, enabling individuals with relatively mild psychological distress, such as depression and anxiety, to easily experience reward effects and opt for gaming as a form of self-medication.

### Application of CBT

Patients with IGD often hold game-related misconceptions, such as “Only in internet games do people recognize me.” These distorted cognitions about gaming contribute to maladaptive behavioral patterns and cravings related to gaming [34]. CBT plays a role in correcting these erroneous beliefs that drive excessive gaming and managing the negative emotions triggered by game withdrawal [35]. Modifying cognitive distortions about gaming can regulate the severity of IGD symptoms and reduce cravings [36]. The application of CBT, originally developed for depression treatment, to various mental health disorders such as PTSD, anxiety disorders, and IGD highlights the shared and distinct features among these conditions and their connection to CBT elements. Therefore, analyzing IGD through the lens of CBT research [37] may provide new perspectives on traditional therapeutic approaches.

**Figure 1:**
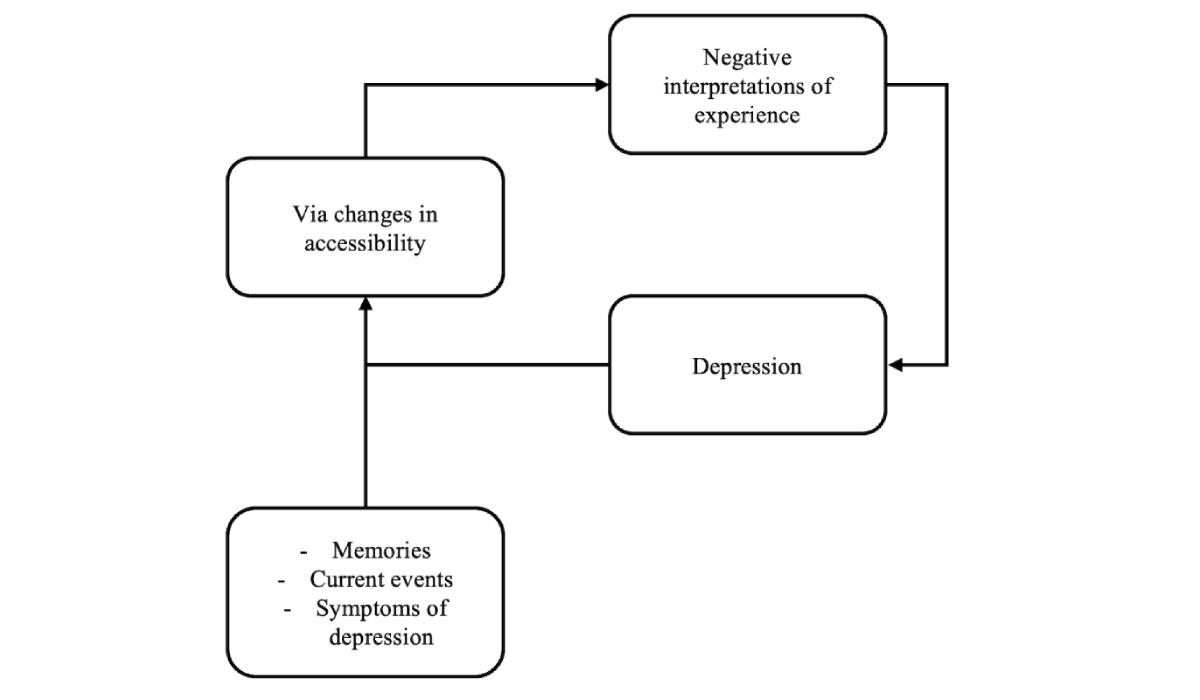
Associative network theory: in associative network theory, human memory, events, and emotions are represented as individual nodes. Each node is interconnected by associative links, and the activation of one node triggers the activation of related nodes.

The theoretical background for the interaction between emotions and cognition in CBT is explained through the associative network theory [38]. CBT involves analyzing and treating the process through which individuals interpret current events based on “automatic thoughts” derived from past experiences. As illustrated in Figure 1, emotions and interpretations interact dynamically, with their sequential relationship reinforcing each other and leading to uncontrollable outcomes [39]. CBT addresses the vicious cycle of psychological distress by introducing alternative thoughts. These newly provided pieces of information compete with negative cognitions during the cognitive processing bottleneck, delaying the processing of negative thoughts and replacing them with alternative perspectives [40].

While associative network theory explains the theoretical basis for the activation of “automatic thoughts,” it has limitations in capturing the complexity of human emotions. For instance, discrepancies may arise between rational beliefs and sensory perceptions. Furthermore, according to associative network theory, the emergence of depressive symptoms requires a triggering event that evokes past experiences and emotions. However, patients with depression often exhibit depressive symptoms not only as a result of current mood and stimuli but also due to an inherent cognitive vulnerability [41,42].

### Application of MBCT

**Figure 2:**
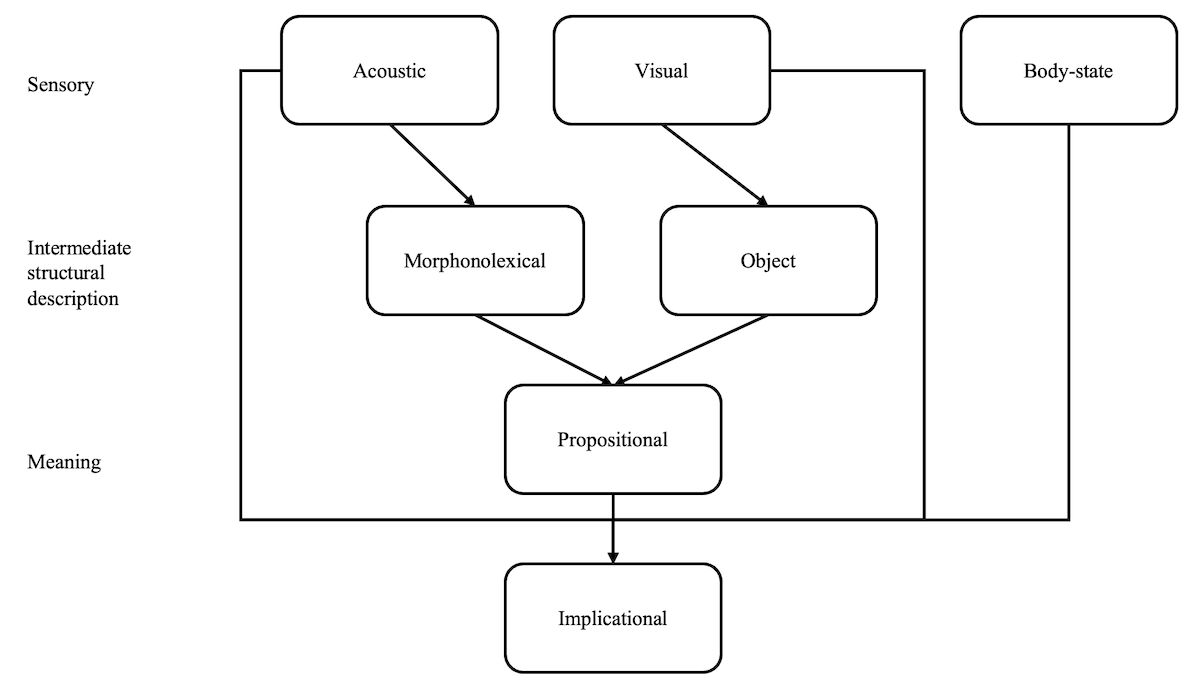
Interactive cognitive subsystem.

**Figure 3:**
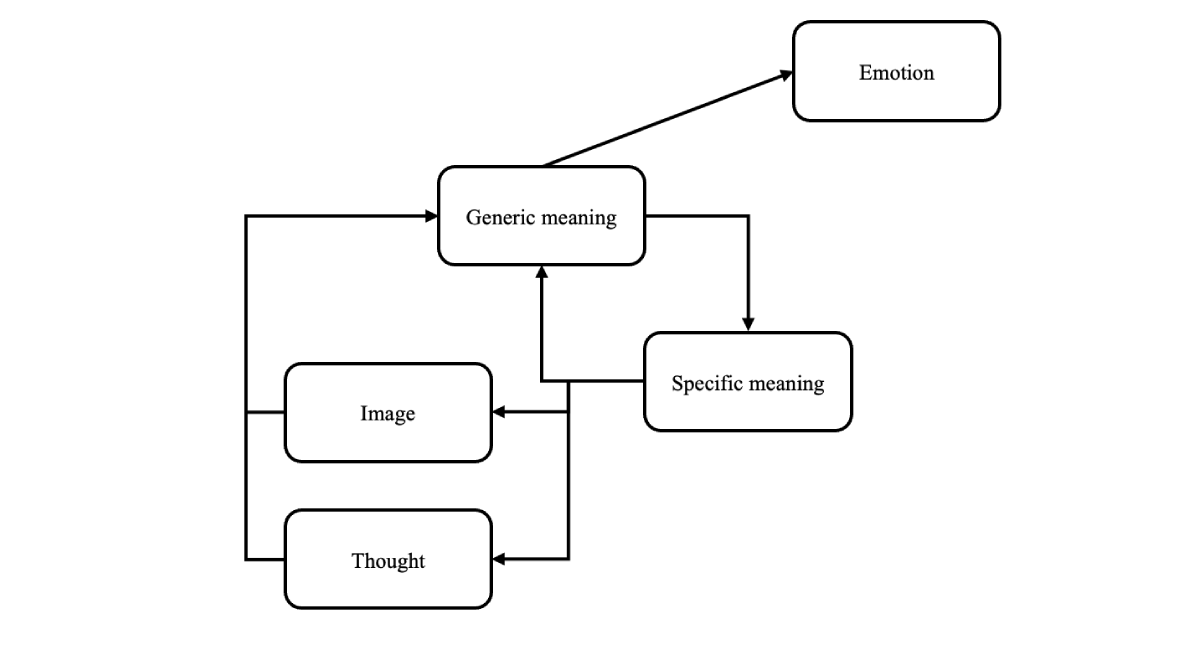
Vicious cycle of the interactive cognitive subsystem: specific meaning influences and reinforces general meaning in a cyclical process.

To explain complex emotions and cognitive vulnerability, the ICS theory has been proposed [43,44]. The model comprises nine subsystems—acoustic, visual, body-state, articulatory, limb, morphonolexical, object, propositional, and implicational—each responsible for processing specific types of information. Through their interactions, cognition and emotion emerge. Visual and auditory information are first interpreted semantically in isolation and then integrated into a specific meaning within the propositional subsystem. This general meaning is further integrated with body-state, visual, and auditory information, evolving into implicational meaning [40]. As illustrated in Figure 2, the interaction between cognition and emotion demonstrates that external stimuli and emotions are indirectly connected. Emotions are portrayed not as mere reactions to external stimuli but as outcomes reflecting cognitive characteristics, thereby explaining why individuals exhibit different responses to stressful situations. Figure 3 illustrates the process where specific meaning and general meaning influence and reinforce each other [43].

MBCT, based on the principles of the ICS, integrates the core mechanisms of CBT with stress-reduction techniques derived from mindfulness meditation. Originally developed for the treatment of recurrent depression, MBCT has demonstrated its efficacy over the past several decades [45-48]. Beyond depression, its effectiveness has been reported for other mental health conditions, including anxiety disorders [49-51], insomnia [52,53], bipolar disorder [54], panic disorder [55], chronic pain [56], cancer-related pain [57], and stress [58].

Key components such as mindfulness skills, depressogenic cognition, self-compassion and cognitive reactivity, and meta-awareness and decentering have been identified as factors associated with or mediating symptom reduction following MBCT [59]. In addition, MBCT has received significantly high ratings in patient self-assessments and has been shown to improve subscales such as “concerning danger” and “controllability of thoughts” [60].

The mindfulness meditation component of MBCT aims to stabilize thoughts by fostering a cognitive process of accepting present thoughts as internal experiences while shifting attention to external experiences by recognizing body-state cues rather than reacting to automatic thoughts. This dual focus promotes emotional regulation and cognitive stability [61].

Nevertheless, several studies have reported no statistically significant differences between MBCT and CBT [60,62,63]. MBCT appears to demonstrate distinct therapeutic effects compared with CBT in patients who have experienced three or more episodes of depression recurrence [64]. However, these effects also seem to be short-term [60]. Despite previous research, the theoretical mechanisms underlying MBCT remain unclear [59].

The limitations of CBT and MBCT are particularly evident in newly defined mental health conditions such as IGD. The authors posit that, unlike the passive mental models described in CBT and MBCT, individuals actively adopt strategies like self-medication to alleviate psychological distress. Therefore, this paper introduces a modified ICS that integrates the self-medication hypothesis into existing cognitive models. This framework aims to develop a more realistic cognitive model, and the application of this model, referred to as MBCT-G, is explained in the following sections.

## Application of MBCT-G

### Modified ICS

The modified ICS focuses on patients’ coping strategies, which were not emphasized in the original theoretical mechanisms. For example, a student with public speaking anxiety may experience situations where they “go blank,” “cannot think of anything,” or “feel mentally frozen” when required to speak in front of a large audience. While these responses may not be ideal, they can be understood as a form of self-medication strategy to cope with the stress of public speaking. By adopting such a strategy, the student may avert their gaze from the audience, quickly read from their script, or even leave the podium without remembering how they spoke, thereby managing the stress in the short term. However, in the long term, this strategy may reinforce their fear of public speaking, lower their self-esteem, and be perceived as an inadequate coping mechanism.

When discussing the mechanisms of cognition and emotion, it is essential to address not only the generation of emotions triggered by external and internal stimuli but also the resulting “reaction” that follows. In the previous example, the experience of giving a presentation is not limited to the emotions felt during the presentation itself but is significantly influenced by the memories and emotions generated during reflection after the situation has ended. Specifically, if an individual evaluates their coping strategy as sufficient and positive after the event, the previously generated emotions can be influenced and transformed into more positive ones. Therefore, the interpretation of emotions should not be confined to a single moment in time; instead, it must account for the distorted recollections formed through self-evaluation of the appropriateness of coping strategies, influenced by past experiences and emotions, as well as the emotions experienced in the present.

For instance, recalling negative experiences such as “feeling darkness before one’s eyes,” “not being able to think of anything,” or “having a blank mind” during public speaking not only reinforces thoughts of psychological distress but also shapes negative self-assessments of the coping strategies used. These negative evaluations, in turn, intensify the negative emotions associated with the experience.

**Figure 4:**
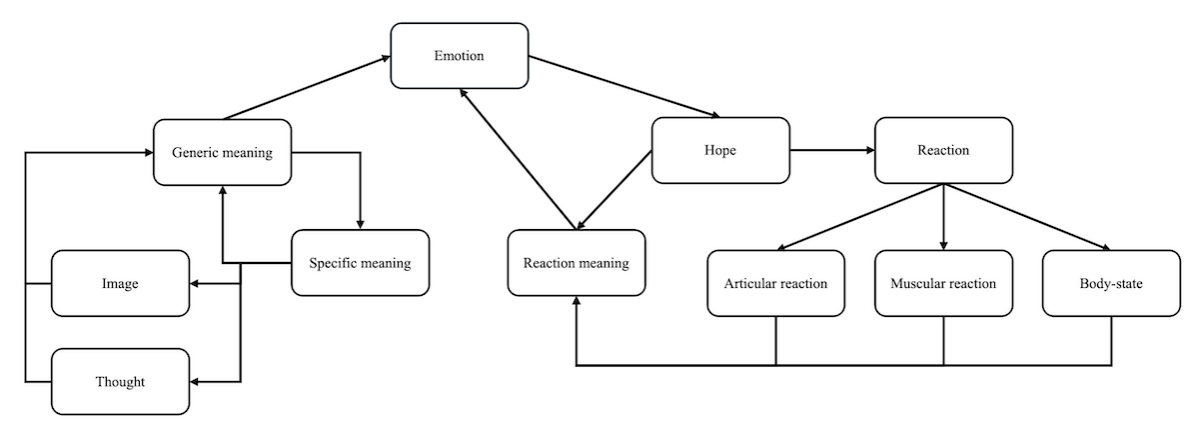
Modified interacting cognitive subsystem.

Figure 4 expands the ICS by incorporating the principles discussed earlier. The additional structure on the right in the modified ICS represents coping strategies and recollections related to emotions.

“Hope” refers to the desired strategy for coping with emotions generated in response to a stimulus. For instance, a student fearful of public speaking might think, “I want to finish my presentation quickly and leave,” “I hope my turn gets skipped,” or “I wish no one pays attention to my presentation.”

“Reaction” represents the active coping mechanism employed in response to “Hope.” In the given situation, since the external change (eg, skipping the student’s turn) is beyond the student’s control, they may choose an active strategy like “Let’s finish the presentation quickly and step down.”

“Reaction meaning” reflects the individual’s self-evaluation of their chosen coping strategy. After adopting the strategy of finishing the presentation quickly, the student might evaluate themselves either positively or negatively. The outcome of this self-assessment is interpreted in the context of their fundamental “Hope” (eg, “I want to finish quickly”), which, in turn, influences the preceding emotions.

In simpler terms, if the strategy of speaking quickly satisfies the initial “Hope,” the preceding “Emotion” will also be alleviated. As a result, the student may continue to rely on the “quick speech strategy” in future similar situations. On the other hand, if the strategy fails, the student might explore alternative strategies. For example, they may choose an active coping mechanism like “I want to relieve stress by playing a game.” If the student successfully alleviates stress through gaming, the previously experienced “Emotion” is resolved, making gaming a preferred coping strategy in similar future scenarios.

In summary, these differences arise because the subjective interpretation of the emotional coping strategy referred to as “reaction meaning,” creates a vicious cycle that influences the emotions resulting from preceding situations. In addition, past experiences, coping strategies, and emotions, such as “I can relieve stress through gaming,” serve as materials for interpreting current emotions, as in “If I play games, my current psychological distress will be alleviated.” To reiterate, experiences, emotions, and coping strategies must be understood as part of a unified principle that unfolds across the flow of psychological time. This interpretation is particularly valuable in disorders where symptoms manifest in behavioral forms, such as IGD, gambling disorder, obsessive-compulsive disorder, and SUD. It offers a foundation for developing new therapeutic approaches.

### How Can Games Be Used in Mental Counseling?

As previously mentioned, the gaming behavior of individuals with IGD can be interpreted as a coping strategy for managing stress resulting from external stimuli. Incorporating the self-medication hypothesis, which is often cited as a cause of SUD, the authors propose the self-prescription hypothesis as a potential explanation for the origins of behavioral addictions like IGD.

In the modified ICS, such coping strategies are effective, at least in the short term, in alleviating psychological distress (commonly referred to as a “reward”), serving as a driving force for the repetition of behavioral addictions. From this perspective, traditional treatments that focus solely on correcting maladaptive thoughts about gaming and reducing gaming time are often ineffective because they fail to provide alternative strategies for managing stress [65].

Treatments that do not offer alternative strategies may lead to symptom relapse or the development of other problematic behaviors, as the underlying cause—stress—remains unresolved. On the other hand, mindfulness meditation can serve as an effective alternative strategy. Activities like meditation and walking can help alleviate stress, reducing the perceived need for internet gaming. However, while these strategies are effective, they may not be sufficient for everyone. Some individuals may lack interest in mindfulness meditation, find it unhelpful in directly addressing their stress, or even perceive it as boring.

To address these limitations, three principles for effective psychological counseling are introduced:

1. Helping patients identify their automatic thoughts: This is the foundational principle of CBT, MBCT, and MBCT-G. Automatic thoughts are the key to uncovering the root causes of stress and form the basis of the therapeutic process. Recognizing these thoughts allows patients to understand the triggers for their psychological distress.
2. Providing alternative strategies to address the sources of stress: As previously discussed, coping strategies influence preceding emotions and play a critical role in stress alleviation. Without appropriate alternative strategies, mental health conditions may persist, worsen, or manifest in different forms of problematic behavior. Effective counseling must present actionable and relevant alternatives tailored to the individual’s needs.
3. Ensuring the proposed alternative strategies are actionable and sustainable: Alternative strategies must be feasible, effective, and realistic for the patient. They should also offer long-term benefits, mindfulness effects, and ease of implementation. For instance, teaching a patient with public speaking anxiety how to “give a good presentation” may not be effective in all situations. These individuals may lack confidence in their presentation skills, but their anxiety might also stem from other causes such as interpersonal relationships, voice quality, or appearance. Simply exposing them repeatedly to presentation scenarios may not be a viable strategy. Moreover, if the patient finds the alternative strategy burdensome or rejects it, they are unlikely to sustain it, leading to cognitive strain during the therapeutic process.

By adhering to these principles, counseling can better address the complexity of individual needs and provide meaningful, sustainable solutions for stress management and mental health improvement.

MBCT-G is a therapeutic approach that incorporates the three principles outlined above. Games serve as a novel medium for experiencing virtual environments that are otherwise unattainable, making them one of the most accessible coping strategies for adolescents and young adults. While the accessibility of games can make even those with mild psychological distress susceptible to addiction, it paradoxically offers an ideal environment for learning healthy coping strategies.

The gaming environment allows individuals to repeatedly practice alternative strategies to address the sources of stress. Because this practice occurs in a virtual setting, it imposes lower cognitive demands. In addition, games can provide diverse scenarios tailored to symptoms and individual needs, while maintaining a consistent therapeutic framework. Furthermore, the gaming environment enables the collection of real-world data during the treatment process and sustains motivation for continued participation in therapy. This combination of features makes MBCT-G a compelling and effective approach to integrating mindfulness and cognitive therapy principles into an engaging and accessible medium.

Several studies have explored the use of games to address conditions such as IGD [66,67], depression [68], anxiety disorders [69], stress [70], attention-deficit/hyperactivity disorder [71], and executive function [72]. However, despite increasing interest in digital therapeutics and game-based treatment methods, few have been recognized by regulatory agencies for their therapeutic efficacy.

In the case of previous research on IGD, most studies have been limited to usability research without control groups [73]. The limitations of previous game-based studies include (1) a lack of understanding of cognition, emotion, and coping strategies, (2) an absence of defined reward mechanisms in games and corresponding alternative strategies, and (3) a poor understanding of game design quality.

A notable previous study using games to treat IGD is Room2Respawn, developed based on mindfulness-oriented recovery enhancement (MORE) [66]. MORE is a therapeutic approach that integrates three psychological counseling techniques: mindfulness, CBT, and positive psychology. It was originally designed for patients with chronic pain who are prescribed opioid analgesics [74]. The same research team investigated the efficacy of MORE in individuals with IGD. In a randomized controlled trial targeting patients with IGD, MORE was found to reduce the severity of the disorder indirectly by correcting maladaptive gaming-related cognitions [36]. However, while Room2Respawn, developed using MORE, assessed the subjective usability of the intervention, it did not demonstrate the efficacy of implementing MORE techniques through gamified software.

## How to Develop MBCT-G

### Overview

In this paper, MBCT-G does not focus on a single game scenario or production method but addresses the overall factors to consider when using games for the treatment of mental health disorders. Therefore, the components of MBCT-G may differ from those of traditional MBCT.

For instance, MBCT for Life, an adaptation of MBCT designed for practical use in everyday life over an 8-week program, comprises the following sessions: (1) waking up from autopilot, (2) another way of being, (3) gathering the scattered mind, (4) recognizing reactivity, (5) allowing and letting be, (6) responding skillfully, (7) how can I best take care of myself? and (8) mindfulness for life [75]. While MBCT is a highly effective tool for alleviating stress, it requires the patient’s active commitment to treatment. As a result, adherence may decrease in patients with cognitive impairments or low motivation for therapy.

The primary focus when designing game-based therapeutic interventions is to improve treatment adherence and provide opportunities for practicing alternative strategies. The key principles to consider when developing specific MBCT-G programs are outlined below.

### Elements of MBCT-G

#### Fun

The first value of therapeutic approaches using games is fun. Educational games available on the market often emphasize the “educational” aspect while lacking gaming elements [76]. This tendency is influenced by factors such as the demands of purchasers (eg, parents), researchers’ negative biases toward games, and a lack of understanding of gamification [77]. Contradictory game designs that disregard learners’ characteristics such as gender and age can hinder therapeutic outcomes [70]. More fundamentally, fun is the most critical reason for applying gamification. Therefore, if concerns arise about the potential adverse effects of “fun games,” researchers should reconsider the project from the outset.

#### Accessibility

Accessibility is a core value of digital therapeutics, including games. As mentioned earlier, in the process of selecting self-prescription methods to address psychological distress, the most important factors are “alleviating psychological distress” and “ease of access.” From this perspective, internet-based games can serve as attractive tools for both the onset and treatment of disorders. Considering the rapid growth of mobile game platforms [78], excessive environmental or time-related constraints on games should be carefully addressed.

#### Reality Projection

Providing an environment where patients can project themselves onto MBCT-G is a topic to consider during the design phase. MBCT-G aims to help patients identify automatic thoughts, correct distorted beliefs about past and present events, and learn healthy coping strategies through experiences and lessons in the virtual world at specific points during gameplay. A game design that fails to reflect reality risks obscuring its therapeutic effects. For example, walking meditation is a core mindfulness practice, but merely watching a character walk on a screen would not yield therapeutic benefits for the patient. To project reality, an intersection between virtual data and real-world information is necessary. This could be as simple as incorporating the patient’s personal information into the game or as complex as creating a sense of connection between the virtual character and the patient’s physical body [79].

#### Discovery of Automatic Thoughts

Identifying distorted beliefs is a critical process in cognitive therapy, and its importance is equally valid in MBCT-G. However, even for professionally trained therapists, uncovering automatic thoughts can be challenging. Therefore, we can use various strategies. First, providing assurance of anonymity can reduce hesitation in sharing personal information [80]. In addition, counseling can be conducted using characters familiar to the patient [81]. Research into new artificial intelligence (AI) technologies is needed to uncover hidden meanings based on counseling content, and offline psychological counseling can be integrated to achieve accurate diagnoses.

#### Experiencing Automatic Thoughts

Experiencing automatic thoughts is one of the distinctive features of MBCT-G. Traditionally, psychological counseling has primarily relied on verbal communication. However, games allow for the direct discovery of distorted beliefs rooted in past experiences. With advancements in AI, various technologies mimicking human society have recently been developed [82]. A key characteristic of these technologies is their ability to form humanlike relationships through the exchange of information and emotions among in-game agents. Researchers, through unpublished studies, have demonstrated that by combining AI technologies with detailed counseling data, past experiences that contributed to psychological distress can be recreated within a game. By leveraging AI, patients are given the opportunity to directly experience distorted beliefs through the reactions of agents that vary depending on the patient’s coping strategies.

#### Experiencing Alternative Strategies in Games

Even when healthy coping strategies to alleviate psychological distress are recommended through counseling, many barriers exist before patients can take actionable steps. Digital technologies, especially virtual reality, provide an environment where new strategies can be vividly explored with minimal effort. For instance, while activities like traveling have often been suggested as accessible alternative strategies during psychological counseling, numerous obstacles have prevented patients from putting such advice into action. In such cases, games can allow for an indirect experience of travel, and if this virtual travel helps alleviate psychological distress, it could serve as motivation for patients to adopt stress-relief strategies through real-world travel. The critical point is that the experience of alternative strategies in MBCT-G should seamlessly connect to real-life applications. The ability to experience alternative strategies is one of the fundamental differences between traditional psychological counseling and MBCT-G.

#### Opportunities for Reflection on Insights

Despite the aforementioned elements, the gamified features of MBCT-G may pose a risk of patients becoming overly immersed in entertainment aspects, potentially overlooking the therapeutic elements. Therefore, MBCT-G should consider providing opportunities for patients to reflect on the lessons and experiences they learn during gameplay through mindfulness meditation.

#### Group Therapy

Group therapy offers advantages such as allowing patients with similar conditions to share their experiences and deepen their understanding of their illnesses. Its effectiveness has been demonstrated across various conditions [83,84]. However, challenges in forming and maintaining groups make physical group therapy sessions difficult to implement [85]. Digital technologies provide diverse ways to facilitate group therapy, both directly and indirectly. For instance, augmented reality technologies like Niantic’s Pokémon GO enable patients to communicate indirectly by creating personal experiences and thoughts in the form of agents on a map [86].

**Figure 5:**
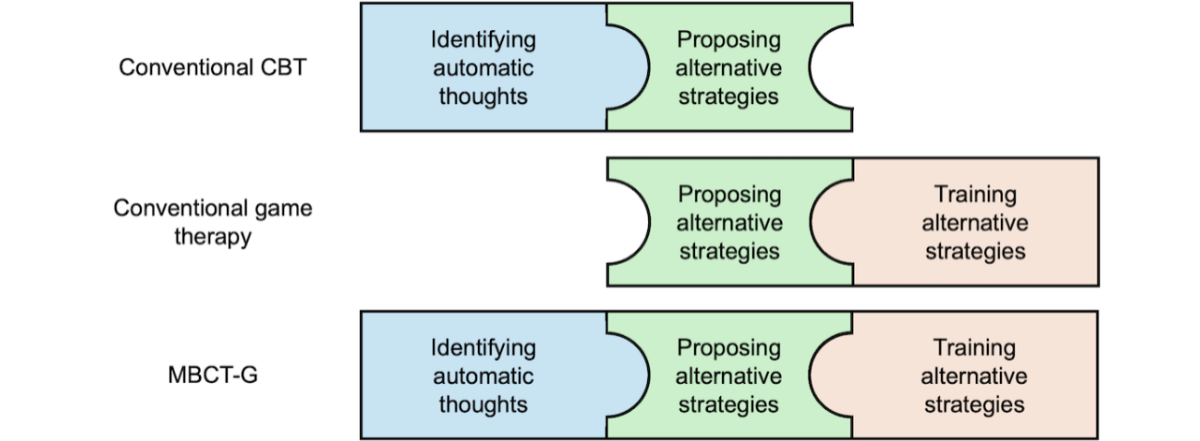
Comparison between MBCT-G and traditional treatment methods. CBT: cognitive behavioral therapy; MBCT-G: mindfulness-based cognitive therapy–game.

The differences between traditional therapies and MBCT-G are compared in Figure 5. Traditional CBT methods are primarily language-based, and while they can propose alternative strategies, they do not provide opportunities for patients to practice these strategies during the treatment process. Existing game-based therapies allow patients to have various experiences in virtual environments; however, they often follow standardized therapeutic procedures, neglecting individual characteristics [87,88]. Also, an example of MBCT-G developed using the 8 elements of MBCT-G can be found in Multimedia Appendix 1.

### Therapeutic Goals and Evaluation of MBCT-G

Internet games, in themselves, cannot be universally labeled as harmful, and merely reducing the time spent playing games may not correlate strongly with the disease’s pathology [89]. Therefore, the primary goal of MBCT-G is to restore significant impairments in daily life, as subjectively perceived by patients and their surrounding individuals.

This goal can be assessed using various methods, including the widely adopted “IGD-20 Test” and other established research tools [90]. In addition, comorbid conditions frequently associated with IGD, such as depression [91], anxiety disorders [92], and attention-deficit/hyperactivity disorder [93], should also be evaluated. Since MBCT-G focuses on the alleviation of subjective symptoms, assessments based on the patient’s counseling history are particularly important.

Advances in AI offer the potential to evaluate disorders using both verbal and nonverbal information [94-96]. AI-based analytic insights should be provided to psychological counselors to facilitate a complementary relationship between technology and human expertise. Furthermore, measuring the frequency and duration of MBCT-G usage is essential for assessing treatment adherence.

Finally, evaluations should include attempts and applications of alternative strategies. These evaluations should be approached from two perspectives:

1. Active engagement with alternative strategies within MBCT-G.
2. Implementation of strategies practiced in the virtual environment in the real world.

Critically, assessing whether patients are adopting healthy coping strategies outside the game environment is the most important therapeutic goal of MBCT-G.

## Five Stages of MBCT-G

### Overview

**Figure 6:**
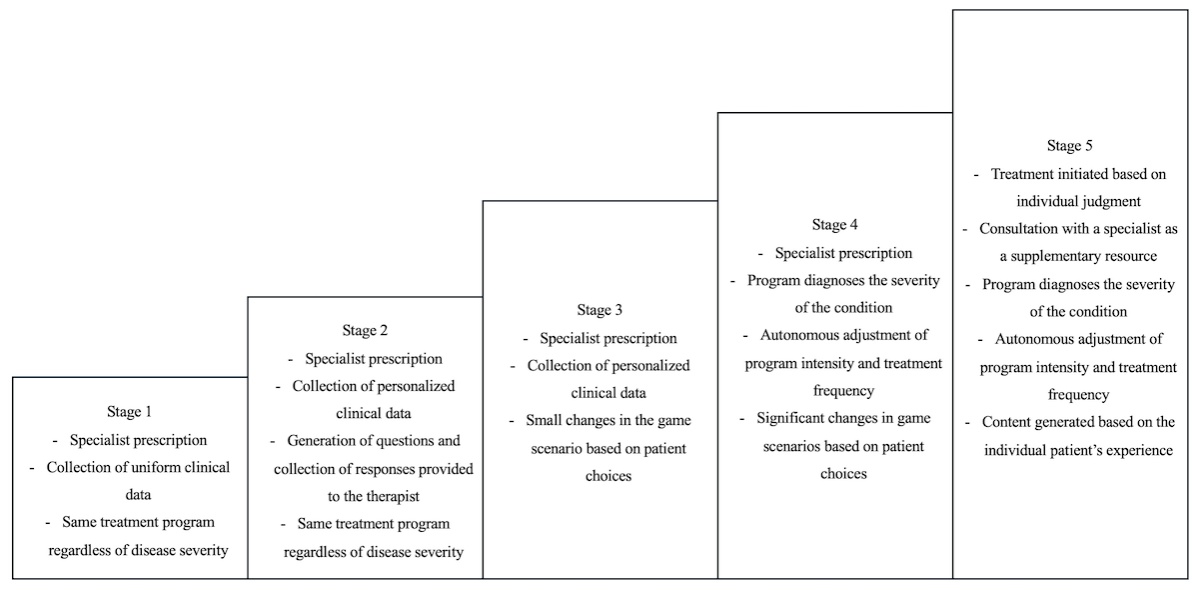
The 5 stages of mindfulness-based cognitive therapy-game.

Finally, the 5 stages of MBCT-G are explained. With technological advancements, new technologies such as autonomous driving, AI, and virtual reality are emerging. MBCT-G aims to create scenarios where patients can practice alternative strategies in a virtual environment, tailored to their treatment motivation and the causes of their condition. However, technological complexity and legal regulations pose obstacles to realizing these strategies. Nonetheless, to advance MBCT-G, it is important to start small and progressively implement these strategies according to the current level of technology (see Figure 6).

### Stage 1

In the first stage of MBCT-G, the patient visits a hospital, receives a prescription from a psychiatrist, and begins treatment. While clinical data are collected through the MBCT-G program, the program’s difficulty level and scenarios are adjusted by the specialist during the hospital visit.

### Stage 2

In the second stage, the MBCT-G program generates personalized questions based on the clinical data collected in stage 1 and asks the patient these questions. Although all patients collect the same clinical data in stage 1, from stage 2 onward, the program gathers tailored clinical data specific to each patient.

### Stage 3

In the third stage, the MBCT-G program diagnoses the severity of the condition in a limited capacity and modifies the game scenarios based on the patient's choices to a restricted extent.

### Stage 4

In the fourth stage, the MBCT-G program independently diagnoses the severity of the condition and adjusts the intensity of the program and the frequency of sessions. Patients can observe significant changes in the game scenarios based on their choices, allowing them to immerse themselves more realistically and practice alternative strategies.

### Stage 5

In the final stage, the MBCT-G program autonomously creates or reconstructs scenarios based on the clinical data collected from the patient. The patient repeatedly trains in virtual reality to address the situations that cause gaming disorder. In stage 5, both diagnosis and treatment are autonomously conducted by the program, with the psychiatrist intervening only in exceptional situations.

## Summary

This paper describes the theoretical background and components of a novel approach called MBCT-G for treating IGD. To achieve this, it elaborates on the conceptual expansion of the self-medication hypothesis, originally proposed as one of the causes of SUDs, and integrates it with the cognitive models of CBT and MBCT. The self-prescription hypothesis and modified ICS presented in this paper can be interpreted as efforts to maintain a form of mental homeostasis.

Homeostasis refers to the property of maintaining a stable and consistent internal environment. Typical examples of homeostasis include maintaining a constant body temperature and regulating the osmotic pressure of the blood. In situations where stress arises due to internal or external stimuli, the human body adjusts factors such as heart rate, respiration, blood pressure, and blood sugar levels to sustain homeostasis [97].

The conceptual terms presented in this paper require further validation in clinical settings. First, previous studies on the relationship among the reward effects, reduced self-control, and craving, as described in the self-medication hypothesis, yield varying results depending on the experimental design [98,99]. Furthermore, the ICS is merely one of several explanations of cognitive structures. These discrepancies stem from the ongoing debate—akin to the “chicken or the egg” question—caused by the diverse comorbidities and complications associated with mental disorders. What is clear, however, is that traditional psychological counseling techniques have been ineffective for behavioral addiction disorders like IGD, underscoring the need for novel therapeutic approaches and theoretical foundations. To address these challenges, this paper proposes the conceptual therapeutic approach and rationale of MBCT-G, based on previous studies and clinical experience.

The concepts introduced in this paper may not necessarily be entirely new. CBT and MBCT emphasize that the present is interpreted based on past events and emotions. Previous research briefly explains the “vicious cycle” where body states, such as trembling hands, a hunched posture, or facial grimacing, change according to emotions, and these changes, in turn, affect emotions [43]. As shown in Figure 2, active behavior and body states influenced by emotions can also affect thoughts and feelings, a notion aligned with the ideas behind MBCT-G. However, in situations of psychological distress, patients’ active behavioral strategies are far more critical than previous studies suggest. Despite this, discussions about such ideas are sparse and have not been incorporated into psychological counseling techniques. Thus, there was a need to emphasize active behavioral strategies in response to psychological distress, which had previously been overlooked, and to incorporate these strategies into the therapeutic process [100].

Theories about the motivation for internet gaming are diverse and extend beyond the self-prescription hypothesis. Internet gaming is known to be driven primarily by the motivations of “escape” and “competition,” which serve as key mediating factors and are reported to directly or indirectly influence the symptoms of IGD.

**Figure 7:**
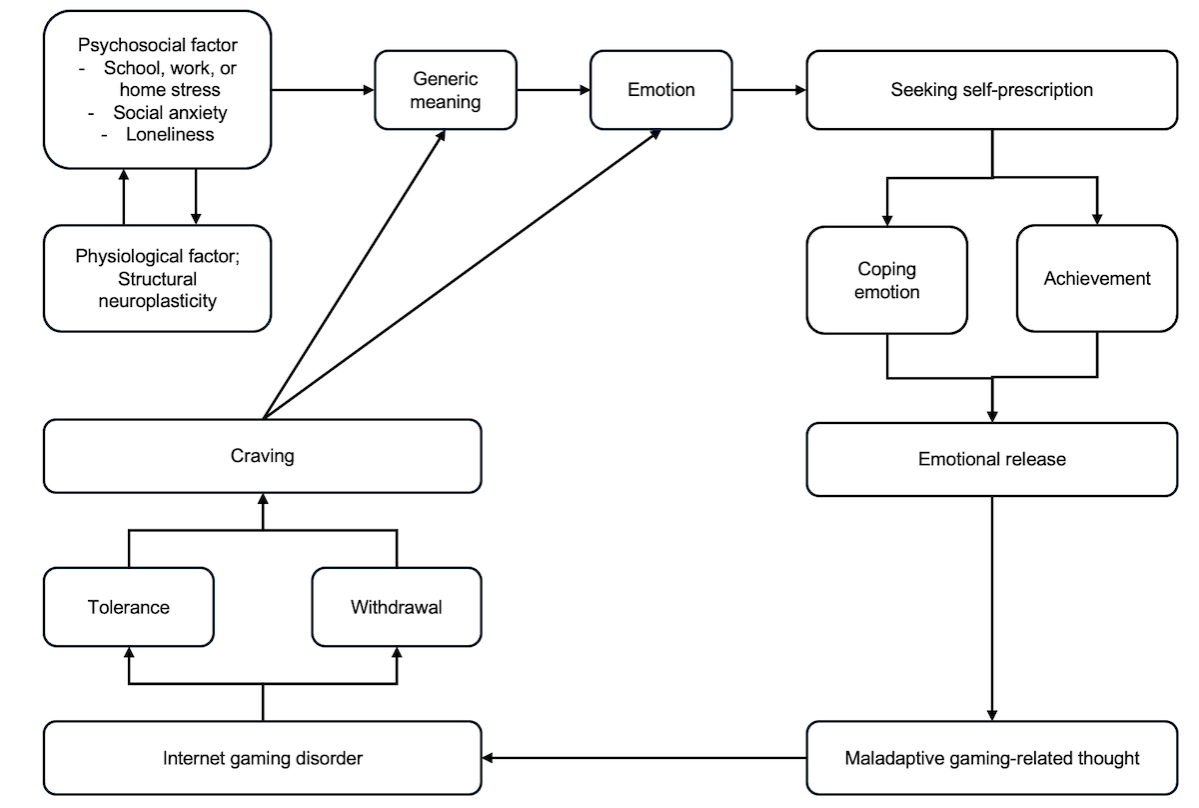
The development process of internet gaming disorder: the tolerance and withdrawal characteristics associated with internet gaming trigger cravings for gaming.

Figure 7 is a visualization of the development process of IGD. Maladaptive thoughts about gaming can arise from various causes. Patients with IGD can be differentiated based on factors such as self-esteem, achievement, and coping strategies [101]. This differentiation suggests that the reasons for gaming addiction are diverse, including “enjoyment of the game itself,” “goal achievement,” and “stress relief.” Therefore, the self-prescription hypothesis proposed in this paper may have limitations in analyzing all motivational factors. However, the various grounds for the self-prescription hypothesis related to the characteristics of IGD and the need for new psychological counseling techniques highlight the importance of continued research in this area.

Digital therapeutics refer to medical approaches that use software and hardware to prevent, treat, and manage physical and mental illnesses. This new approach to disease management and treatment offers several advantages. First, it allows patients to receive treatment without the need to visit hospitals or counseling centers, providing freedom from time and space constraints. Second, online mental health treatment can eliminate the stigma and negative perceptions that patients might otherwise face. Third, because sessions can be conducted without the involvement of a physician or counselor, it is easier to monitor treatment adherence, and the approach is cost-effective [73]. Games are a type of digital therapeutic method, and the enjoyment they provide can be particularly effective for patients lacking motivation for treatment. This is especially true for adolescents, who exhibit the highest prevalence of IGD and simultaneously have a strong affinity for games and digital devices, making game-based approaches highly effective.

Considering that patients with IGD often choose gaming as the most effective means of alleviating psychological distress, teaching them to repurpose gaming, which has been used inappropriately, into a tool for positive life outcomes may ironically prove to be the most effective therapeutic method.

## Conclusion

IGD affects 3% of the population, and with technological advancements, more individuals are at risk of developing this condition. However, there is currently no clear treatment available. IGD is not a primary disease but rather a result of “self-prescription” in response to emotional stressors. Therefore, unlike traditional mental health treatments that focus on the disorder itself, it is essential to provide alternative activities that can alleviate negative emotions. This paper introduces MBCT-G as an alternative activity. MBCT-G is a program designed to explore alternative activities through gaming, focusing on the reaction and reward processes, areas that traditional treatments have not emphasized. This paper serves as the first theoretical foundation for the development of MBCT-G. MBCT-G is necessary as a method to provide alternative emotional relief strategies for treating self-prescription disorders such as IGD.

## Appendix

Multimedia Appendix 1 An example of mindfulness-based cognitive therapy–game.

## Abbreviations

AI: artificial intelligence
CBT: cognitive behavioral therapy
DSM-5: Diagnostic and Statistical Manual of Mental Disorders, Fifth Edition
ICD-11: International Statistical Classification of Diseases and Related Health Problems, 11th Edition
ICS: interacting cognitive subsystem
IGD: internet gaming disorder
MBCT-G: mindfulness-based cognitive therapy–game
MORE: mindfulness-oriented recovery enhancement
PIU: pathological internet use
PTSD: posttraumatic stress disorder
SUD: substance use disorder
WHO: World Health Organization
