# Supplementary material for: Mindfulness-Based Cognitive Therapy–Game: An Ironic Way to Treat Internet Gaming Disorder
Source: J Med Internet Res. 2025 Mar 27;27:e65786. doi: 10.2196/65786 (PMC11986384; doi:10.2196/65786)
Supplement: Multimedia Appendix 1 [file jmir_v27i1e65786_app1.docx]

# Appendix

## Example of MBCT-G: A Small-Scenario

Stanford Smallville is a study that demonstrates the possibility of characters using LLMs (Large Language Models) to form relationships and influence each other. In this virtual town, characters implement plans and actions through processes of memory, perception, and recall. The series of processes presented in this study resembles the relationship between emotions and actions defined in Figure 4. The figure below illustrates the structure of “A small-scenario” agent based on Modified Interacting Cognitive Subsystems.


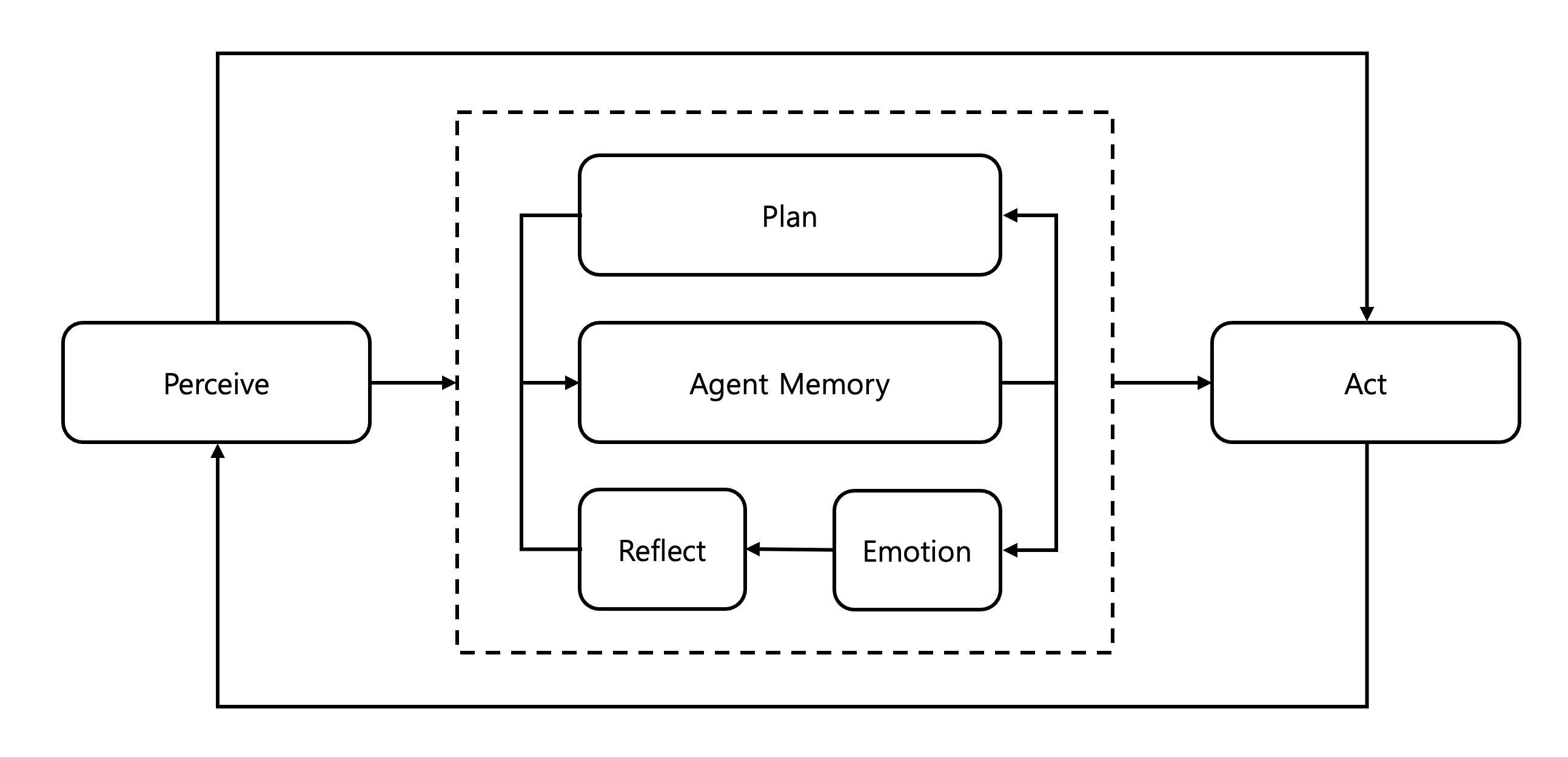


Structure of LLM agent

The most notable feature of Stanford Smallville is that the agents modify their plans and actions based on their interactions with each other. This advantage can be applied to the principles of CBT (Cognitive Behavioral Therapy), where faulty beliefs are corrected by allowing patients to reconsider their automatic thoughts from a different perspective.

In the technique proposed by this paper, the patient first works with a psychological counselor to explore the specific situations that trigger negative emotions. The counseling session covers people, places, objects, and past memory related to feelings of depression or anxiety. Next, using the data on the people, places, and utterances mentioned during the first session, along with LLMs, the situation triggering the negative emotions is recreated in a virtual environment called “Small-Scenario.” The characters and environments in the game reflect the subjective people and places from the patient’s memory.

During the game therapy stage, the patient freely controls a character that represents themselves over a game period of 1–2 days, interacting with other characters. The primary goal of this process is for the patient to express emotions such as anger, depression, and sadness toward the source of their stress while simultaneously accepting those emotions. The goal of the program is to demonstrate, through repeated gameplay, that the patient’s active thoughts and behavioral changes can influence the surrounding characters and environment, leading to different outcomes than before. The patient can resolve their issues through “different choices from the past,” “conversations with others,” and “alternative activities to cope with stress.” This allows the patient to discover their automatic thoughts and learn positive coping strategies for handling situations. Thanks to Stanford Smallville being open-source, we are able to utilize this remarkable technology to develop MBCT-G. A game that reflects the patient’s past experiences provides motivation for immersion in a virtual environment. Additionally, by leveraging 3D graphic engines in combination with Stanford Smallville, which was developed as a 2D game, MBCT-G can offer a more realistic training experience for alternative activities beyond internet gaming.

## Doctor Agent

In Small-Scenario, the agents are broadly classified into the patient, characters, and the doctor. The characters are further divided into hostile characters, positive characters, and bystanders. Among these, the “doctor” is a very special agent that plays a key role in setting the overall direction of the game. The doctor agent performs three main roles: first, it assists by exploring faulty beliefs together with the patient through conversation. Second, it serves as a guide in stressful situations, helping the patient choose alternative coping strategies. Lastly, it controls the actions and speech of other agents to ensure they remain appropriate and therapeutic within the overall scenario.

While agents powered by LLMs have the strong advantage of behaving freely like real people, they also have limitations that make them difficult to use as medical devices due to hallucinations. The doctor agent in Small-Scenario is trained with rule-based machine learning, which forces the LLM-trained agents to act according to the direction of the scenario. In other words, when the patient’s speech is analyzed and it is determined that they are executing alternative coping strategies well, other agents are commanded to engage in speech or actions that produce positive outcomes. Rule-based machine learning ensures predictable results, allowing the program to function as a medical device, while LLMs can overcome the limitations of traditional medical devices by creating a virtual environment that aligns with the user’s thoughts. Within this structure, the hallucination drawback of LLMs can be mitigated by considering different pathways, particularly when dealing with unpredictable human relationships. The doctor agent repeatedly asks the patient “questions” throughout the scenario. For example, when the doctor agent detects that the patient is experiencing conflict with a character, it doesn’t make arbitrary judgments but instead confirms this through natural questions. Based on the data collected from the patient’s responses, the doctor agent adjusts the direction of the scenario and influences the behavior of other agents. Therefore, rather than relying solely on the accuracy of the LLM, this approach satisfies the stability required for a medical device by working as a strategy centered on natural questions in Human-AI interaction. Figure below provides an overview of the entire framework.


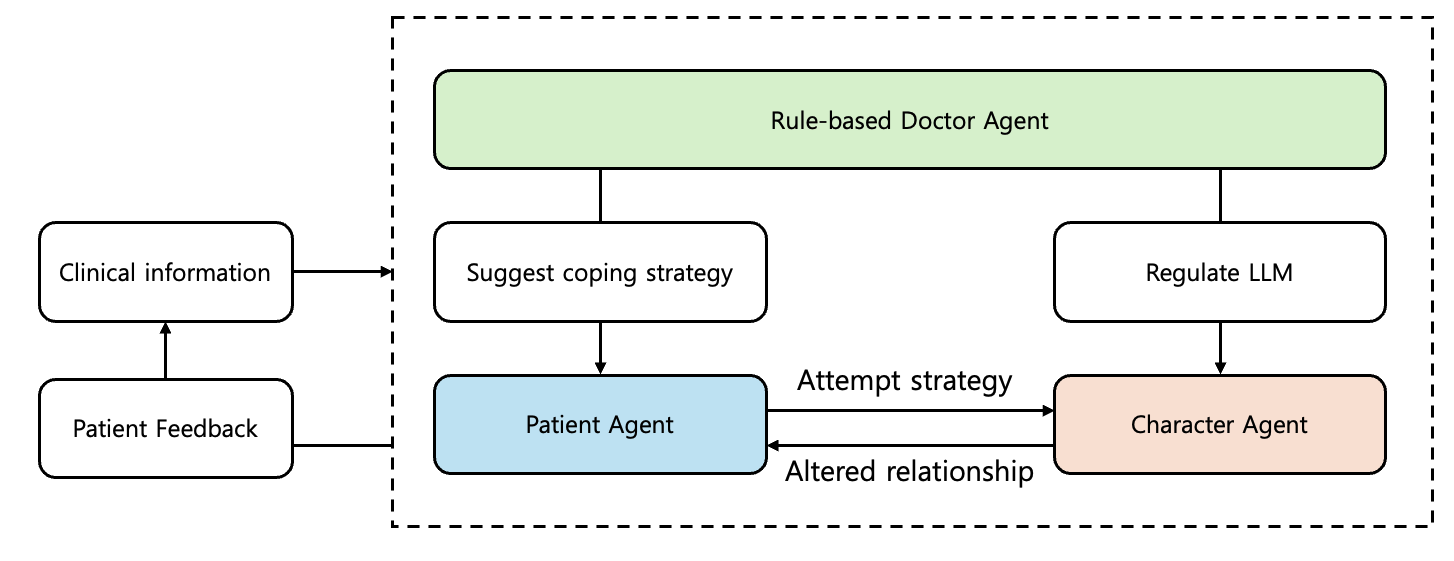


Schematic diagram of Small Scenario

The patient provides feedback on how natural the characters and environment felt at the end of the scenario.​
